# Identification of Nontuberculous Mycobacteria Species Isolated from Water Samples Using Phenotypic and Molecular Methods and Determination of their Antibiotic Resistance Patterns by E- Test Method, in Isfahan, Iran 

**Published:** 2012

**Authors:** Sharareh Moghim, Ensieh Sarikhani, Bahram Nasr Esfahani, Jamshid Faghri

**Affiliations:** 1 *Department of Microbiology, School of Medicine, Isfahan University of Medical Sciences, Isfahan, Iran*

**Keywords:** Antibiotic resistance patterns, E-test, hsp65, NTM, Water samples, 16S rRNA

## Abstract

**Introduction:**

Many studies have shown epidemiological links between strains isolated in tap water, and those isolated from patients. Molecular methods linked to PCR are more reliable and faster for identification of non- tuberculous mycobacteria (NTM). In this study molecular methods were used for identification and typing of NTM.

**Materials and Methods:**

Five hundred ml of 85 water samples was passed through 0.45 μm filters. The filters were transferred directly onto 7H10 Middle Brook solid media, containing 15% OADC. PCR for 16S rRNA was done and the PCR product (1500 bp) was sequenced. PRA of the *hsp65 *gene was investigated to identify the species of isolates. For evaluation of susceptibility of NTM to antimycobacterial agents, E-test method was used.

**Result:**

The genus of 26 isolated NTM was confirmed by 16s rRNA sequence based method. Nineteen isolates of Mycobacteria were differentiated using *hsp*65 genes PRA. The dominant isolates were *M. fortuitum *(26.7%), *M. chelonae *like organism (13.3%) and *M. mucogenicum *(13.3%). Seventy one percent of NTM species were resistant to isoniazid, 64% to rifampin, 57% to ethambutol, 35% to tetracycline, 14 % to azithromycin and 7.1 % to amikacin.

**Conclusion:**

The results showed that E-test method is not a proper technique for antimycobacterial assay because some NTM species are slow in growing and have no growth on Muller Hinton agar. Regarding the 16S rRNA sequence analysis, the identification of isolates was restricted to the genus level, because 99% similarity within 16S rRNA of two isolates may or may not determine the same species.

## Introduction

Nontuberculous mycobacteria have been found in potable water, chlorinated biofilms, water distribution systems, soil, dust, food, aerosols and even in free living amoeba in water and animal reservoirs. These organisms can cause hypersensitivity, pneumonitis, asthma and bronchitis, infection of skin, wounds and glands. Moreover these infections are serious threats for cystic fibrosis patients. In recent years NTM have been reported to be important agents of infection in immunosuppressive patients (1). NTM can be isolated from different natural sources among them water (1-4). These organisms have been isolated from hard conditions such as low pH and nutrients. Several species of NTM have been identified in different environments including public drinking water, pool, impotable tap water, water cooler etc. Therefore, water may act as an important NTM source for infection transmission to human. In some studies, the presence of NTM in water samples collected from different regions were determined (1-3, 5). Traditionally, NTM has been detected in clinical and environmental samples by culture-based techniques; however these techniques may not be well suited for environmental samples. Indeed, identification of NTM by culture and phenotypic characterization is widely used but it takes 4 to 6 weeks or longer for slow growing species and identification of some species may be missed by biochemical methods. The rapid methods used for identification include high performance liquid chromatography (HPLC), DNA probes, restriction fragment length polymorphism (RFLP) using various target regions including heat shock protein 65 KD gene (*hsp*65), *ITS* and* rpo *B (1, 6, 7). PCR-based molecular methods are faster and more reliable and for identification of NTM (1, 5). PCR restriction fragment length polymorphism analysis (PRA) of *hsp*65, 16S rRNA may help to define difficult taxonomical status to identify NTMs. Identification and taxonomic position of NTMs which remained unresolved by cultural and biochemical characteristics could be partially resolved by PRA and sequencing of *hsp*65, which are frequently used for mycobacterial identification. *hsp*65 gene is used widely for identification of NTM to species level because of its variability compared to some other conserved genes such as 16S rRNA (6-8) but there is no standard pattern for all of species. Therefore, combination of two or more methods is needed for reliable identification. Nonetheless, 16S rRNA sequencing was needed for a definite response in many cases. Mycobacterial susceptibility testing is important for appropriate patient management. It should be done on initial isolates of Mycobacteria and on clinically significant isolates of certain nontuberculous mycobacteria. E-test (AB Biodisk, ) is a new concept for determining MICs of antimicrobial agents. The relative easy use of this method is a feature which is attractive to smaller clinical laboratories that do not have the facilities necessary to prepare complex susceptibility testing media. In this study 16S rRNA was used for identification of NTM in genus level and differentiation of mycobacteria from other acid fast bacteria and PCR-RFLP of *hsp*65 gene was used for typing of NTM species. The MICs of some antimicrobial agents were determined by scaled E-test strips.

## Materials and Methods

***Collection and preparation of the samples***

Eighty five water samples were collected from different sources including mineral water (8.2%), dentistry unit water (10.6%), impotable tap water (14.1%), potable water supply (14.1%), haemodialysis unit water (8.2%), general pools (8.2%), river water (7.1%), water spout (11.8%), cold water dispenser (11.8%) , and water boiler (5.9%) in Isfahan. Water samples were prepared by grab sampling method (9). Total chlorine content was determined using DPD method at sampling locations (9, 10). The water in total volume of 2-litters’ was added to sterile Erlenmeyer flasks, containing 1.8 ml of a 3% solution of sodium thio-sulfate as antichlorine and 0.04% cetylpridinium chloride as antimicrobial agent. Five hundred ml of the samples was passed through 0.45 µm filters. The filters were transferred directly onto 7H10 Middle Brook solid media, containing 15% OADC (oleic acid, albumin, dextrose, catalase). The plates were examined once a week for eight weeks. The acid-fast positive colonies were transferred to Lowenstein- Jensen (LJ) slant media and incubated at 37 ^o^C. 

***Phenotypic identification***

The mycobacterial isolates were identified by the growth characteristics, including growth at 25, 37 and 42 °C, pigment production, semi-quantitative catalase test, Tween 80 hydrolysis, arylsulfatase test (3 and 14 days), heat-stable catalase (pH 7, 68 °C), pyrazin amidase (4 and 7 days), urease, nitrate reduction test and colony morphology. Reference strains of *M. smegmatis (*PTCC 1307*) and M. fortuitum (*ATCC 6841*) *were used as control species in all steps of this study. 

***Evaluation of susceptibility of NTM by E-test method***

E-test strips were used to determine the susceptibility of NTM isolates to amikacin, rifampin, ciprofloxacin, ethambutol, tetracycline, doxycycline, azithromycin and isoniazid antibiotics. The turbidity of the bacterial Middle brook 7H9 broth culture was adjusted with additional sterile distilled water to equal a McFarland 1 turbidity standard. Muller Hinton containing 10% OADC and 500 mg/lit cyclohexamide without glycerol were used for the test. The MIC was determined by the intersection of the inhibition ellipse with the concentration of antimicrobial agent on the E-test strip.

***Genus and species identification of NTM by PCR-RFLP and 16S rRNA sequences analysis***

Chromosomal DNA of NTM isolates were extracted using CTAB (cetyl-trimethyl ammonium bromide) method (11). PCR of the *hsp*65 gene was performed using the forward primer for *hsp*65, Tb11 (5΄ACC AAC GAT GGT GTG TCC AT 3’) and the reverse primer, TB12 (5’ CTT GTC GAA CCG CAT ACC CT 3’), as described previously (12). The PCR products were detected by 1.5% agarose gel electrophoresis. The amplified products of *hsp*65 gene regions were digested with two restriction enzymes, *Hae*III and *BstE*II, according to the recommendations of the manufacturers. The digested products were separated on 10% polyacrylamide gel electrophoresis (PAGE) and the RFLP patterns were analyzed according to fragments sizes (12-15). PCR for 16S rRNA was done in a 50 µl reaction mixture containing 50 mM KCl, 10 mM Tris–HCl (pH 9), 2.5 mM MgCl_2_, 200 μM dNTPs, 1.25 U Taq polymerase, 30 pmol of each primer and 10 ng of DNA template. The sequences of primers were 5´-GGAGAGTTTGATCCTGGCTCAG-3´ as forward and 5´- AAGGAGGTGATCCATCCGCA-3 as reverse (16). Samples were then subjected to one cycle of 96 °C for 5 min, followed by 35 cycles of 95 °C for 40 sec, 68 °C for 30 sec, and 72 °C for 30 sec, and one final cycle of 72 °C for 10 min in Biomerta Gradient thermocycler and an Eppendorf AG 22331 (Germany). PCR products were run on 2% agarose gel and examined for the presence of the amplicon band after ethidium bromide staining. PCR products were purified with spin column kit (Qiagene, Germany) and sent for sequencing (Bioneer, Korea).

## Results

Chlorine concentrations of the samples ranged from 0 to 1.8 mg/l. Parameters related to investigated water samples and the identified NTM is summarized in Table 1. Twenty one isolates were identified by growth characteristics and conventional biochemical tests. Dominant isolates were *M. fortuitum-* (26.7%), *M.* *chelonae* like organism (13.3%) and *M. mucogenicum *(13.3%).

The MICs for 8 current routine antibiotics against mycobacterial infections were examined. Seventy one percent of NTM species were resistant to isoniazid, 64% to rifampin, 57% to ethambutol, 35% to tetracycline, 14% to azithromycin and 7.1% to Amikacin (Table 2). 

**Table 1:** Summary of the frequency of NTM isolated from different water sources

Water sources	Number (%) of samples	Number (%) of positive samples	NTM species
Water supply	12 (14.1%)	5 (41.7%)	*M. gordonae, M. mucogenicum, M. furtuitum ss. fortuitum, M. chitae , M. neoaurum*
Undrinkable tap water	12 (14.1%)	6 (50%)	*M. chelonae, M. furtuitum, * *M. mucogenicum , M. chelonae like organism , 2* *Unidentified* species
Waterspout	10 (11.8%)	2 (20%)	*M. mucogenicum, M. furtuitum ss. fortuitum*
Cold water dispenser	10 (11.8%)	5 (50%)	*M. chelonae *like organism*(2), M. furtuitum 3th variant(2), 1 *Unidentified species
Dentistry unit water	9 (10.6%)	2 (22.2%)	*M. mucogenicum , M. fortuitum 3th variant*
Pool and baths water	7 (8.2%)	2 (28.6%)	*M. chelonae *like organism* , M. duvalii*
Water in haemodialysis center	7 (8.2%)	0	-
Mineral water	7 (8.2%)	0	-
River water	6 (7.1%)	0	-
Hot water dispenser	5 (5.9%)	0	-

**Figure 1:**
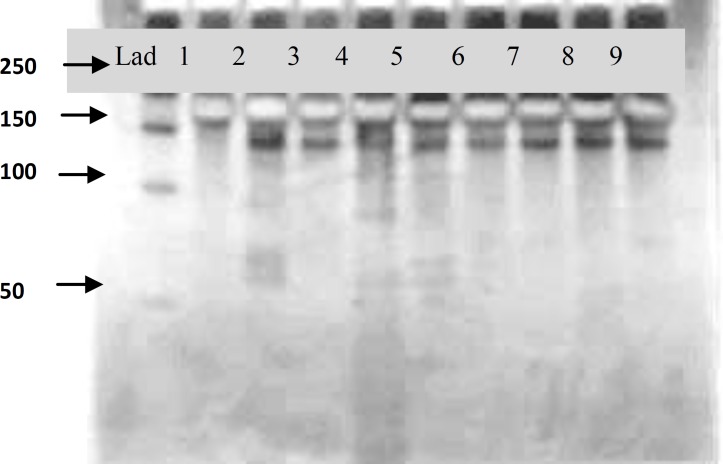
PAGE of *hsp*65 441-bp amplicons of NTM digested by *BstE*II. Lad: DNA ladder (50 bp), lanes: 1: unidentified; 2: *M. gordonae;* 3:* M. gordonae*; 4: unidentified; 5, 6: *M. fortuitum*; 7: *M. kansasii*; 8:* M. furtuitu;*and 9*: M. smegmatis *(PTCC 1307)

PCR-RFLP of the *hsp*65 gene was examined to identify the species of isolates. A 441 bp fragment of *hsp*65 genes was amplified and digested by *BstE*II and HaeIII. The digested fragments separated on 10% PAGE and RFLP patterns were analyzed according to fragment sizes (17). Nineteen isolates of Mycobacteria were differentiated using *hsp*65 genes PCR-RFLP. Three isolates could not be identified at the species level. Some of the patterns of digested *hsp*65 PCR products are shown in Figure 1. The obtained sequences results of 16S rRNA were compared to those available in GenBank*.* All groups of isolates showed a similar score lower than 99%.

**Figure 2:**
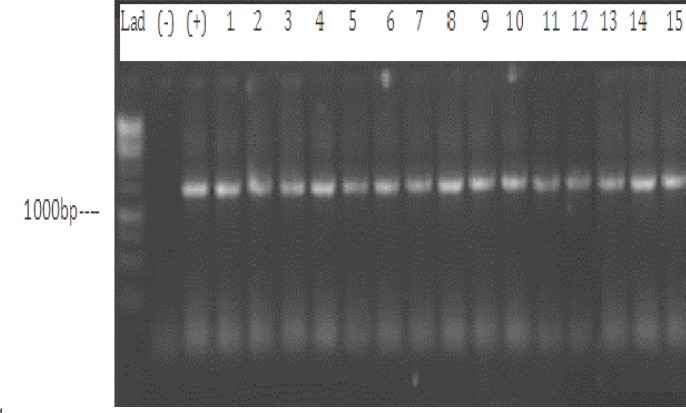
Agarose Gel Electrophoresis of 16S rRNA amplified gene. Lad: 1000 bp Ladder, (-): Negative control, (+): Positive control (*M. smegmatis*), lane 1-15: 16S rRNA PCR products

## Discussion

In this study, 85 water samples were collected from different sources. The prevalence of NTM was determined using culture, biochemical tests, 16s rRNA sequencing, and PCR-RFLP analysis of *hsp*65 gene. 25.9% of water samples contained one to three different species of NTM. Twenty one species were identified by conventional methods. Dominant isolates were* M. fortuitum* (26.7%), *M.* *chelonae* *like organism* and *M. mucogenicum *(13.3%). The results shown that there was not any correlation between the species of mycobacteria in water samples and total chlorine concentrations (*P* value> 0.05) but the correlation with temperature was species-related (*P *value< 0.05%). Covert and Rodgers isolated different species of NTM from 54% of ice samples and 35% of public drinking water (5). Shin *et al* showed that half of tap water samples in hospital environment are positive for mycobacteria (22). Argueta *et al* reported that 25 of 121 food samples (20.6%) were positive for NTM (20). The results of this study showed that the incidence of NTM was similar to other geographical environments.

**Table 2:** The MICs (μg/ml) of conventional antimycobacterial drugs against NTM isolates by E test method

Antibiotics	AK	RI	CI	EB	TS	DC	AZ	IZ
Mycobacterium sp. *M. chelonae* like organism	0.125	R	0.75	0.064	0.032	0.094	0.5	R
*M. chelonae*	0.5	1.5	0.19	R	0.006	0.064	0.5	R
*M. chelonae* like organism	1.5	0.002	0.25	R	0.023	0.047	R	R
*M. mucogenicum*	0.19	R	0.125	1	R	0.047	1.5	R
*M. chelonae* like organism	1	R	0.03	R	R	8	4	R
*M. duvalii*	3	R	0.19	R	0.125	1	2	R
*M. mucogenicum*	0.25	0.125	0.38	R	0.002	0.016	1.5	R
*M. smegmatis*	0.125	R	0.047	0.094	0.002	0.064	0.5	8
*M.fortuitum*	R	2	4	R	0.5	6	R	R
*M.smegmatis*	2	12	0.023	R	R	3	2	R
*M.fortuitum*	8	R	0.75	R	R	0.023	8	R
*M. flavescense*	128	R	0.023	0.5	R	0.5	128	0. 25
*M. fortuitum*	8	R	0.094	0.19	0.38	0.125	8	64
*M. chelonae like organism*	0.5	R	1	0.032	0.094	256	0.5	2
*E. coli* (ATCC 25922)	2	4	0.064	R	R	1	64	R

R: completely resistant NTM without any susceptibility zone

IZ: isoniazid; RI: rifampicin; EB: ethambutol; AZ: azithromycin; AK: amikacin; CI: ciprofloxacin; DC: doxycycline; TS: tetracycline

In this study, a 441-bp fragment of NTM *hsp*65 gene was amplified and digested by *BstE*II and *Hae*III and their patterns were analyzed on polyacrylamide gel. As shown in Figure 1, there were different PCR-RFLP profiles. Nineteen isolates (86.4%) of NTM were identified at species level by PRA. Three isolates presented profiles that were different from the known RFLP profiles and did not correspond with other studies (8, 12-15, 20-22). Turenne *et al* showed that *hsp*65 PRA is useful for identification of some species such as *M. gastri *and* M. kansasii* that cannot be identified by other methods (16). Wong and Yip, reported that PCR-RFLP targeting *hsp*65 gene region could identify 74.5% of NTM (21). Telenti *et al* identified 10 NTM isolates to the species level using PCR-RFLP in which 439 bp PCR products were digested with BstEII and HaeIII (12). Absence of standardization for all of NTM species may cause some confusing in pattern analysis especially in new species. On the other hand, the interpretation of bands is ambiguous for highly polymorphic species (8). In this study sequencing of 16S rDNA harboring constant region (to identify mycobacterium at the genus level) was used. This method is faster and easier than other molecular methods; however, some disadvantages include base errors and ambiguous bases that may lead to misleading results.

## Conclusions

We conclude that 16Sr RNA sequence database analysis is controversial for NTM identification. Regarding the 16S rDNA sequence analysis, a similarity percentage of 99% or higher between two isolates defines a similar species. Indeed, two isolates with a 99% similarity in their 16S rDNA may or may not belong to the same NTM species. According to the results of E- test, resistance of NTM species to rifampin, isoniazid and ethambutol was more than 57%. It seems that rifampin, isoniazid and ethambutol as single drug is not useful for treatment of NTM infections although, ciprofloxacin and doxycycline have more effect. Doxycycline was effective in lower dose than ciprofloxacin and azithromycin. In conclusion using of doxycycline, amikacin, ciprofloxacin, tetracycline or combination of them could be more suitable for treatment of NTM infections.
